# Pituitary tumor-transforming gene expression is a prognostic marker for tumor recurrence in squamous cell carcinoma of the head and neck

**DOI:** 10.1186/1471-2407-6-242

**Published:** 2006-10-09

**Authors:** Christine Solbach, Marc Roller, Frank Eckerdt, Silke Peters, Rainald Knecht

**Affiliations:** 1 Department of Gynecology and Obstetrics, Johann Wolfgang Goethe University School of Medicine, D-60590, Frankfurt am Main, Germany; 2 Department of Otolaryngology, Johann Wolfgang Goethe University School of Medicine, D-60590, Frankfurt am Main, Germany; 3 Business Unit Oncology, Novartis Institutes for Biomedical Research, CH-4057, Basel, Switzerland; 4 Department of Pharmacology, University of Colorado School of Medicine, Denver, CO 80262, USA

## Abstract

**Background:**

The proto-oncogene pituitary tumor-transforming gene (PTTG) has been shown to be abundantly overexpressed in a large variety of neoplasms likely promoting neo-vascularization and tumor invasiveness. In this study, we investigated a potential role for PTTG mRNA expression as a marker to evaluate the future clinical outcome of patients diagnosed with primary cancer of the head and neck.

**Methods:**

Tumor samples derived from primary tumors of 89 patients suffering from a squamous cell carcinoma were analyzed for PTTG mRNA-expression and compared to corresponding unaffected tissue. Expression levels were correlated to standard clinico-pathological parameters based on a five year observation period.

**Results:**

In almost all 89 tumor samples PTTG was found to be overexpressed (median fold increase: 2.1) when compared to the unaffected tissue specimens derived from the same patient. The nodal stage correlated with PTTG transcript levels with significant differences between pN0 (median expression: 1.32) and pN+ (median expression: 2.12; *P *= 0.016). In patients who developed a tumor recurrence we detected a significantly higher PTTG expression in primary tumors (median expression: 2.63) when compared to patients who did not develop a tumor recurrence (median expression: 1.29; *P *= 0.009). Since the median expression of PTTG in patients with tumor stage T1/2N0M0 that received surgery alone without tumor recurrence was 0.94 versus 3.82 in patients suffering from a tumor recurrence (*P *= 0.006), PTTG expression might provide a feasible mean of predicting tumor recurrence.

**Conclusion:**

Elevated PTTG transcript levels might be used as a prognostic biomarker for future clinical outcome (i.e. recurrence) in primary squamous cell carcinomas of the head and neck, especially in early stages of tumor development.

## Background

Pituitary tumor-transforming gene (PTTG) was originally identified in rat pituitary tumors by differential display PCR. Stable transfection in mouse NIH-3T3 fibroblasts induced cellular transformation *in vitro *and led to tumor formation in athymic nude mice [1]. Further functional analysis identified PTTG as human securin, the functional analogue to the Pds1p protein of budding yeast and the Cut2p protein of fission yeast [2]. In yeast, the securins Pds1p and Cut2p bind to separins (Esp1p or Cut1p respectively), thereby inhibiting sister chromatid separation until degradation of securins by the anaphase promoting complex (APC) at the metaphase to anaphase transition. The release of active separins leads to the degradation of a distinct protein of the multi-protein complex which holds sister chromatids together since DNA duplication in S-phase [3]. Furthermore, as confirmed by preliminary investigations in mammalian cells, the expression levels of PTTG transcripts and protein are upregulated in rapidly proliferating cells and in a variety of solid tumors including carcinomas of the lung, breast, melanomas, hematopoietic neoplasias and tumor cell lines [4,5]. Moreover, PTTG-protein levels fluctuated during the cell cycle, reaching a peak in mitosis [6,7].

Worldwide, more than half a million new cases of squamous cell carcinomas of the upper aerodigestive tract are annually diagnosed. Up to 90% of these individuals are inhaling tobacco smoke and/or ingest alcohol on a regular basis [8]. Most of these patients are introduced to clinical care with an UICC (Union International Contre Cancer) stage III or IV, and show an overall life expectancy of less than 40%, despite multimodal treatment strategies like surgery, radiation and chemotherapy [9]. Currently, the most important prognostic factor of head and neck squamous cell carcinoma (HNSCC) is the pathological stage, especially the nodal stage (pN). Due to the fact that tumors of the same stage can show different biological behaviors resulting in different prognoses, we focused on new biological factors to improve the tools for treatment and prognosis of HNSCC. Presently, a set of biological parameters for prognostic evaluation of HNSCC has been described such as p53, epidermal growth factor receptor, cyclin D1 and Polo-like kinase mRNA and protein expression, which we found recently as independent markers for the survival of patients with HNSCCs [10-12]. Considering the probabilities of tumor recurrences in early stages of tumor development (pT1/2N0M0), most of these parameters do not suffice for predicting clinical outcome. Recent findings suggest that the expression of pituitary tumor transforming gene (PTTG) might serve as a marker for tumor invasiveness in colorectal cancer [13] as well as a marker for lymph node invasion of breast cancer [14] and small and non small cell lung cancer [15]. Based on these observations, we investigated the prognostic value of pituitary-tumor transforming gene (PTTG) expression in squamous cell carcinomas of the head and neck.

In the present study, we examined the PTTG mRNA expression of a large number of HNSCC tumors and compared those to PTTG mRNA expression levels in microscopically unaffected mucosa of the same patients. Interestingly, we were able to detect elevated PTTG mRNA levels in the vast majority of HNSCC and more importantly, PTTG levels were related to the tumor stage. In summary, on the basis of a 5-year observation period, PTTG mRNA expression in the primary tumor improves the prediction of tumor recurrence especially in early stages of tumor development.

## Methods

### Patients and tumor samples

Analyses were performed on tumor samples of 89 patients with HNSCC (78 male and 11 female) who were treated at the Department of Otolaryngology of the J. W. Goethe-University, School of Medicine, Frankfurt am Main, Germany from 1987 to 1997. Patients were diagnosed with either oropharyngeal carcinomas (n = 66) or larynx carcinomas (n = 23). The median age was 54 years with a range from 22–84 years. All patients were smokers and ingested alcohol on a regular basis. Staging of patients prior to treatment included computed tomography scan of the head und neck, chest x-ray and sonography of the neck and abdomen as well as endoscopy of the upper aerodigestive tract, oesophagoscopy and bronchoscopy. All 89 patients underwent a complete resection of the primary tumor and unilateral elective neck dissection of the clinically negative neck. In case of clinically positive lymph nodes, unilateral or bilateral neck dissection was performed, depending on the involvement of lymph nodes. All neck dissections were carried out as modified radical neck dissections type III [16]. Vital tumor probes were removed from the primary site and microscopically normal tissues were taken approximately 5 cm apart from the tumor margins. Tissue samples were immediately frozen in liquid nitrogen and stored at -80°C. In line with histological criteria, all tumors were classified as HNSCCs, WHO grading I-III. The post surgical stage of each patient was classified in accordance to the tumor-node-metastasis system. Most patients were diagnosed as stage IV (T3-4N2M0, n = 36; T3-4N3M0, n = 19), while other patients were stage III (T3N1M0, n = 9), stage II (T2N0M0, n = 15) and stage I (T1N0M0, n = 10). Patients of stages I and II were submitted to surgery alone, patients of stages III und IV underwent surgery and postoperative radiotherapy. Standard radiation schedule was used with 2 Gy fractions given as once-a-day treatment of a total dose of 70 Gy applied to the primary region and the neck. Chemotherapy was given nonconcomitantly, applying three cycles of cisplatinum (20 mg/m2/day) and 5-fluorouracil (1000 mg/m2/day), according to a standard schedule [17]. Tumor recurrences were treated chemotherapeutically with the same regimen until no response or progressive disease was detected in two dimensions by computed tomography scan. Routine investigations were conducted over a period of 5 years post therapeutically, which involved clinical investigation, sonography and computed tomography of the head and neck as well as sonography of the abdomen and chest x-ray. The majority of patients, who suffered from recurrences developed loco regional metastasis (n = 46), while only few patients exhibited lung metastases (n = 3). The median time to recurrence was 8.5 months after treatment with a range between 2 and 61 months. Survival time of terminal patients ranged from 1–80 months with a median of 15.5 months. The median quality of life index during the observation period with reference to the Eastern Cooperative Oncology Group scale (ECOG) was 3. The hospital local ethics committee approval was obtained for the study.

### RNA-isolation and northern blot analysis

#### Tumor sample preparation

Tumor samples were stored in liquid nitrogen immediately after excision. A sample of 500 – 1000 mg was homogenized in a guanidinium isothiocyanate solution. RNA was prepared by ultracentrifugation at 125.000 g through a 5.7 M CsCl2 cushion. 2 μg total RNA were separated in a denaturing 1.5% agarose/2% formaldehyde gel, RNA quality was confirmed by ethidium-bromide staining before RNA was transferred to nylon membranes which were subsequently probed with digoxigenin labeled PTTG full-length cRNA probe.

#### Probes for northern blotting

A reverse-transcription PCR using Expand Reverse Transcriptase according to manufacturers instructions was performed (all solutions and enzymes for PCR protocols were obtained from Roche). The cDNA template was used in a standard PCR with a sequence-specific 5'-primer (5'-CAG AAT GGC TAC TCT GAT CTA TGT TGA TAA G-3') and the specific 3'-primer linked to T-7 polymerase promoter (5'-TAA TAC GAC TCA CTA TAG GGG CCC AGC TGA AAC TT-3'; underlined T-7 promoter sequence) using Expand High Fidelity PCR System. The probes were generated using the PCR product after sequencing as template for transcription by T-7 polymerase with digoxigenin labeled dUTP.

#### Hybridization and detection of RNA products

The prehybridizytion/hybridization steps were performed at 68°C for three hours/over night in DIG Easy Hyb solution (Roche). The membranes were washed twice under high stringent conditions in 0.1 × SSC/0.1% SDS and products subsequently detected by immunological methods using alkaline phosphatase conjugated anti-DIG-Fab fragments and chemiluminescence reagent CDPStar according to the manufacturers protocol (Roche). After detection, membranes were stripped and reprobed with β-Actin mRNA Probe (Roche).

#### Detection of mRNA expression level and statistics for tumor samples

The intensity of the specific chemiluminescence signal was quantified using Kodak 440 CF documentation system running the 1D image analysis software. A rectangle was drawn around the specific signal and intensity was calculated by subtracting the mean of the background signal (Kodak, Rochester, NY). The RNA load was normalized to *β-Actin *and the quotient *(density units *PTTG)/*(density units β-Actin) *was used for the statistical analyses as described previously [10]. All analyses were performed using the software package SPSS 9.0 for Windows.

### Immunohistochemistry

Antigen retrieval was performed on 4 μm tissue sections by boiling in 0.01 Mol citrate buffer (pH 6.0) for 20 minutes. Sections were incubated with polyclonal rabbit anti-PTTG (Zymed) (or rabbit preimmuneserum for control) for 60 minutes at 37°C, subsequently washed in Tris- buffered saline (TBS) pH 7.4 and incubated with alkaline phosphatase labeled mouse anti rabbit antibody (DAKO), followed by neufuchsin detection (DAKO). All sections were counterstained with hematoxylin.

## Results

### PTTG mRNA is overexpressed in squamous cell carcinomas of the head and neck

**Figure 1:**
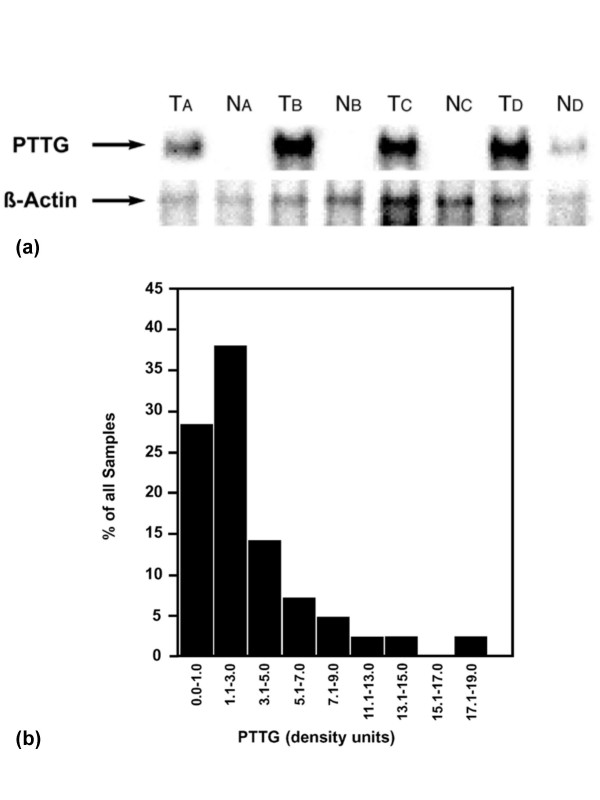
**Expression of PTTG mRNA in HNSCC tumor samples**. Total RNA (2 μg) underwent electrophoresis, transferred to positively charged nylon membranes and probed with PTTG specific probe (TA = T2N0M0/no recurrence, TB = T4N1M0/recurrence, TC = T3N2M0/no recurrence, TD = T2N0M0/recurrence, N indicates the corresponding normal tissue). Bottom of panel (a) shows the corresponding *β-Actin *signal after stripping and re-hybridization procedure. (b) Distribution of normalized PTTG expression levels in all (n = 89) analyzed tumor samples.

Northern blot analysis revealed that PTTG mRNA was hardly detectable in normal adult mucosa of the upper aerodigestive tract, as well as microscopically unaffected mucosa of tumor patients. In contrast to these unaffected tissue specimens a prominent signal of approximately 0.9 kb, representing the PTTG transcript, was detected in the vast majority of 89 tumors derived from patients with HNSCC, indicating that PTTG mRNA is overexpressed in these specimens (Fig 1a). The normalized PTTG expression ranged from 0.00 up to 18.67 density units (mean: 3.22 d.u.; median: 1.59 d.u.) in tumor tissue samples (Figure 1b) and from 0.00 to 1.56 d.u (mean: 1.23 d.u.; median: 0.74 d.u.) in the corresponding normal mucosa (median fold increase: 2.1 when compared to the unaffected tissue specimens derived from the same patient). The Wilcoxon test for comparison of tumor expression levels versus unaffected normal sample levels had an overall significance of *P *< 0.05 (n = 89).

### Expression levels of PTTG correlate with tumor staging parameters

**Figure 2:**
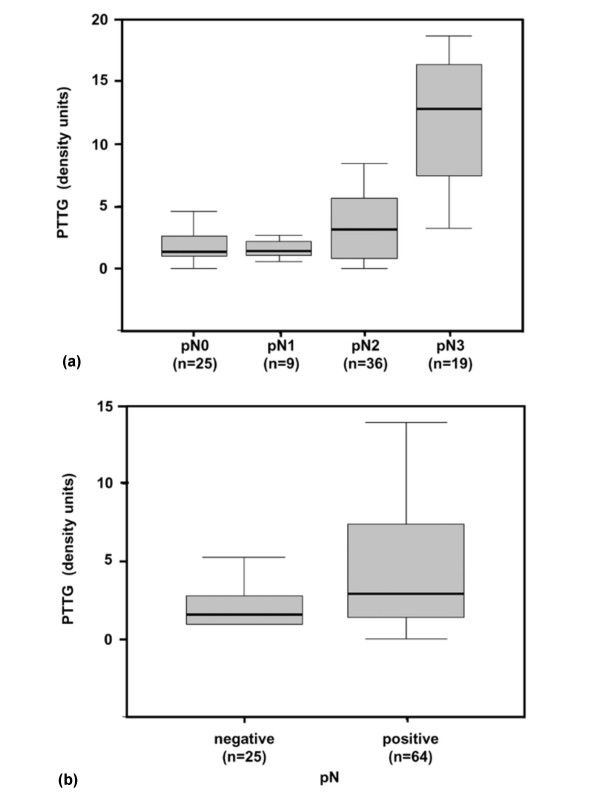
**Distribution of PTTG expression in primary tumors with different pN stages**. Boxplot shows PTTG expression of all analyzed primary tumors (n = 89) grouped to their corresponding pN-stages (a). The pN-stage correlated to PTTG expression level with a significance of *P *= 0.021. Thereby significant (*P *= 0.016) differences between the pN0 (n = 25, negative) and pN+ (n = 64, positive) staged patients could be detected (b).

In order to determine whether PTTG expression levels correlate with clinical parameters, we performed a Kruskal-Wallis analysis, which revealed that PTTG mRNA levels correlate with the pathological T stage (pT; *P *= 0.013, an overview of significant correlations found in this study is given in Table 1). Furthermore, pN-stage, the most important prognosis factor in head and neck cancer, correlated with PTTG expression levels with an overall significance of *P *≤ 0.021 (Figure 2a). Moreover, highly significant (*P *= 0.016) differences between pN0 and pN+ were observed (median fold increase: 2.1) (Figure 2b and Table 1).

### PTTG is an indicator for the risk of tumor recurrence

**Figure 3:**
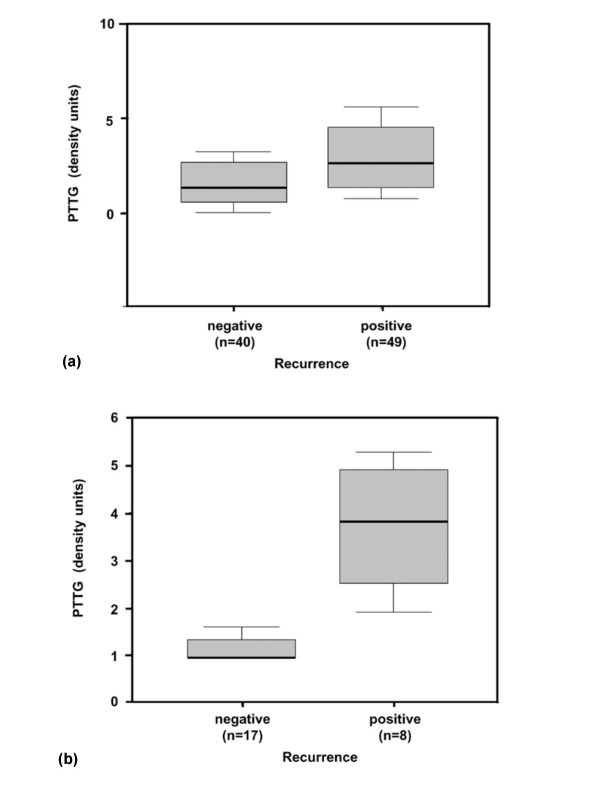
**PTTG mRNA expression in primary tumors of patients with tumor recurrences**. (a) Boxplot shows the PTTG expression in primary tumors of all patients (n = 89) who suffered from a recurrence (positive, n = 49) versus the patients who did not develop a recurrence (negative, n = 40) within the 5-years observation period (*P *= 0.009). Boxplot (b) shows the distribution of PTTG mRNA levels in primary tumors in comparison to those levels in tumors of pN0 (n = 25) staged patients with (positive) and without (negative) a tumor recurrence (*P *= 0.006).

**Figure 4:**
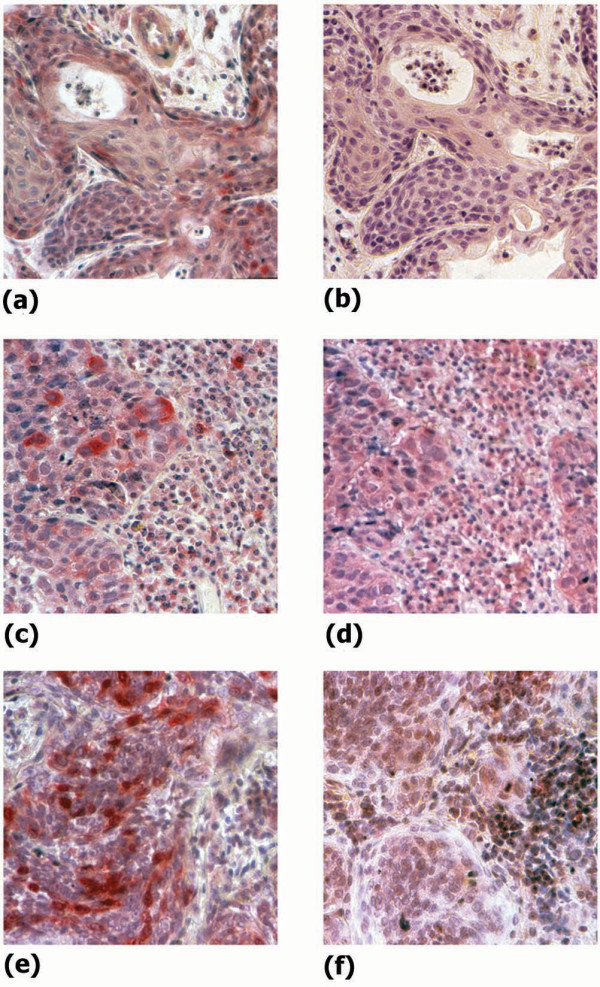
**Immunhistochemical analysis of PTTG**. Exemplary PTTG staining of a primary tumor of a patient (pT1N0M0), who did not develop a tumor recurrence within the observation period (a). Staining of a primary tumor of a patient (pT1N0M0) who developed a tumor recurrence 24 months after primary tumor resection (c). (e) PTTG staining in the tumor recurrence of the same patient. (b, d and f) corresponding control sections after IgG staining.

We next investigated whether PTTG mRNA expression of primary tumors can be correlated with the development of recurrences. Based on an observation period of 5 years after primary therapy, patients who developed tumor recurrences had significantly higher PTTG expression levels in their primary tumors when compared to those patients who did not develop a tumor recurrence (Mann Whitney U test, *P *= 0.009). The median expression level of recurrent tumors (n = 49) was 2.63 d.u., with a range from 0.74 to 18.67 d.u., whereas in non-recurrent tumors, PTTG expression was detectable only within a range of 0.00 to 8.37 d.u. with a median of 1.29 d.u. (Figure 3a). Additionally, by using the median cut off level, we revealed that 68% of the patients exhibiting PTTG = median (1.59 d.u.) developed a tumor recurrence while this was true for only 39% of those patients who exhibited PTTG < median. Moreover, for the clinical more homogenous pN0 group (n = 25, all Patients were T1/2N0M0 and received surgery as single therapy), the median PTTG expression level of recurrent carcinomas (n = 8) was 3.82 d.u. (range: 1.90 to 5.29 d.u.) in comparison to the median level of 0.94 d.u. (range: 0.00 to 2.54 d.u.) of non-recurrent tumors (n = 17). Since this difference was highly significant (*P *= 0.006), we hypothesized that PTTG expression levels may serve as a prognostic factor to predict different recurrence probabilities within the pN0 population (Figure 3b). This notion was confirmed by using the median cut off level (1.32 d.u.) of PTTG mRNA expression in the pN0 group. In this group, only a single patient (n = 17), whose primary tumor exhibited PTTG expression < median, developed a tumor recurrence. By contrast, all 8 patients from the pN0 group, who showed PTTG mRNA expression values = median in their primary tumors, developed a tumor recurrence. Subsequently, we performed a series of immunohistochemical analyses to further substantiate our findings and to correlate them to clinically more applicable immunohistochemical methods,. Expectedly, for pT1/2N0M0 patients, we could detect a strong immunoreactivity of primary tumors, especially in tumors of patients who acquire a tumor recurrence. In contrast, primary tumor samples from patients, who did not develop a tumor recurrence, exhibited only weak immunoreactivity (Figure 4).

## Discussion

Estimation of prognosis and biological behavior of head and neck cancer is primarily based on clinicopathological parameters, mainly on tumor site and regional lymph node involvement. However, it is well known that tumors of the same clinicopathological stage can behave differently, especially in regard to the risk of tumor recurrences. Therefore, current work is focusing on the evaluation of molecular parameters to differentiate and complete the prognostic statement, which is critical to refine and improve treatment strategies [12]. The pituitary tumor-transforming gene is a recently characterized oncogene, originally isolated by its differential expression in pituitary tumor cells [1]. PTTG mRNA and protein is overexpressed in a variety of carcinomas [4,6,14,15], suggesting that PTTG may be involved in tumorigenesis. In addition to its function as human securin during the cell cycle, recent studies convincingly demonstrate that PTTG acts as a transcriptional activator. This function seems to be regulated by the activation of the mitogen-activated protein kinase cascade (MAPK) by epidermal growth factor (EGF). The interaction of PTTG with activated MAP kinase kinase (MEK-1) is crucial for the transactivation function of PTTG [18]. The MAPK-pathway is known to be important in the regulation of cell growth, apoptosis and differentiation. Numerous studies reported a correlation between over-expression of the epidermal growth factor receptor (EGFR, c-erbB1) and poor prognosis in head and neck squamous cell carcinomas [19]. Moreover, it has been demonstrated that MAPK activation regulates rat PTTG translocation into the nucleus where it is able to transcriptionally activate the oncogene *c-myc *in vitro. [20]. This might be of special interest, because *c-myc *is known to play a critical role in the control of cellular proliferation and deregulation of *c-myc *is associated with a variety of tumors. In human cancer, overexpression of c-myc protein stimulates cell cycle progression, leads to transformation and blocks differentiation [21]. The induction of *c-myc *expression by overexpression of PTTG results in increased cell proliferation and colony formation, strengthening the idea of PTTG playing an important role in the regulation of cell growth [18]. Furthermore, PTTG also induces basic fibroblast-growth factor (bFGF) secretion, indicating a role of PTTG in tumor angiogenesis [13,22,23] that is essentially required for tumor survival. Altogether, these observations underline the role of PTTG in development and maintenance of neoplastic processes.

## Conclusion

In this study, we examined 89 patients with HNSCC and compared the PTTG expression of their primary tumors to unaffected tissues of the same patient. In comparison with normal mucosa of the upper aerodigestive tract and in microscopically unaffected mucosa, we found PTTG mRNA to be overexpressed in the vast majority of HNSCCs. Moreover, the observation that metastatic as well as recurrent HNSCC exhibited a significantly higher PTTG expression than non-metastatic and non-recurrent primary tumors suggests, that determination of the PTTG status might provide feasible means to predict the metastatic potential of a tumor. This is especially of clinical interest in an early clinicopathological stage (N0, pN0), where the determination of PTTG expression in the primary tumor in addition to conventional staging may contribute to evaluate the risk of a tumor recurrence more precisely. In our study, PTTG expression did not correlate to any other standard clinico-pathological parameters or to survival of patients.

Although loss of yeast securin Pds1p or Drosophila securin pimples is lethal [24,25], mice lacking PTTG are viable and fertile. PTTG-/- fibroblasts exhibit dysfunction in the cell cycle [26,27]. Thus, besides its transcriptional regulation of target genes, like *c-myc*, PTTG has essential functions in promoting cell cycle events. Being aware of these observations and given the fact that overexpression of PTTG promotes cellular transformation [1], we suggest that PTTG might represent a promising target to inhibit tumor cell growth, thereby improving treatment strategies for patients with a high probability of developing a tumor recurrence [28].

## Competing interests

The author(s) declare that they have no competing interests.

## Authors' contributions

CS, MR and FE were responsible for the acquisition and interpretation of experimental data required for this study; drafting and revising the manuscript.

RK and MR were responsible for conception and design of the study, performed statistical analyses and interpreted obtained data.

SP and RK were responsible for surgical and clinical acquisition and interpretation of data required for the study.

All authors read and approved the final manuscript.

## Pre-publication history

The pre-publication history for this paper can be accessed here:



## Figures and Tables

**Table 1:** Overview of significant correlations between PTTG overexpression in primary tumors and standard clinico-pathological parameters found.

**Significant correlations**	**Level of significance**
pT	0.013 (n = 89)
pN	0.021 (n = 89)
Tumor recurrence	0.009 (n = 89)
pN0 versus pN+	0.016 (n = 89)
Tumor recurrence in T1/2N0M0 group	0.006 (n = 25)
